# Video-Based Communication Assessment of Physician Error Disclosure Skills by Crowdsourced Laypeople and Patient Advocates Who Experienced Medical Harm: Reliability Assessment With Generalizability Theory

**DOI:** 10.2196/30988

**Published:** 2022-04-29

**Authors:** Andrew A White, Ann M King, Angelo E D’Addario, Karen Berg Brigham, Suzanne Dintzis, Emily E Fay, Thomas H Gallagher, Kathleen M Mazor

**Affiliations:** 1 Department of Medicine University of Washington School of Medicine Seattle, WA United States; 2 National Board of Medical Examiners Philadelphia, PA United States; 3 Collaborative for Accountability and Improvement University of Washington Seattle, WA United States; 4 Department of Pathology University of Washington School of Medicine Seattle, WA United States; 5 Department of Obstetrics and Gynecology University of Washington School of Medicine Seattle, WA United States; 6 Department of Bioethics and Humanities University of Washington Seattle, WA United States; 7 Meyers Primary Care Institute University of Massachusetts Medical School Worcester, MA United States

**Keywords:** medical error disclosure, simulation studies, communication assessment, graduate medical education, crowdsourcing, patient-centered care, generalizability theory, medical education, medical error, communication

## Abstract

**Background:**

Residents may benefit from simulated practice with personalized feedback to prepare for high-stakes disclosure conversations with patients after harmful errors and to meet American Council on Graduate Medical Education mandates. Ideally, feedback would come from patients who have experienced communication after medical harm, but medical researchers and leaders have found it difficult to reach this community, which has made this approach impractical at scale. The Video-Based Communication Assessment app is designed to engage crowdsourced laypeople to rate physician communication skills but has not been evaluated for use with medical harm scenarios.

**Objective:**

We aimed to compare the reliability of 2 assessment groups (crowdsourced laypeople and patient advocates) in rating physician error disclosure communication skills using the Video-Based Communication Assessment app.

**Methods:**

Internal medicine residents used the Video-Based Communication Assessment app; the case, which consisted of 3 sequential vignettes, depicted a delayed diagnosis of breast cancer. Panels of patient advocates who have experienced harmful medical error, either personally or through a family member, and crowdsourced laypeople used a 5-point scale to rate the residents’ error disclosure communication skills (6 items) based on audiorecorded responses. Ratings were aggregated across items and vignettes to create a numerical communication score for each physician. We used analysis of variance, to compare stringency, and Pearson correlation between patient advocates and laypeople, to identify whether rank order would be preserved between groups. We used generalizability theory to examine the difference in assessment reliability between patient advocates and laypeople.

**Results:**

Internal medicine residents (n=20) used the Video-Based Communication Assessment app. All patient advocates (n=8) and 42 of 59 crowdsourced laypeople who had been recruited provided complete, high-quality ratings. Patient advocates rated communication more stringently than crowdsourced laypeople (patient advocates: mean 3.19, SD 0.55; laypeople: mean 3.55, SD 0.40; *P*<.001), but patient advocates’ and crowdsourced laypeople’s ratings of physicians were highly correlated (*r*=0.82, *P*<.001). Reliability for 8 raters and 6 vignettes was acceptable (patient advocates: G coefficient 0.82; crowdsourced laypeople: G coefficient 0.65). Decision studies estimated that 12 crowdsourced layperson raters and 9 vignettes would yield an acceptable G coefficient of 0.75.

**Conclusions:**

Crowdsourced laypeople may represent a sustainable source of reliable assessments of physician error disclosure skills. For a simulated case involving delayed diagnosis of breast cancer, laypeople correctly identified high and low performers. However, at least 12 raters and 9 vignettes are required to ensure adequate reliability and future studies are warranted. Crowdsourced laypeople rate less stringently than raters who have experienced harm. Future research should examine the value of the Video-Based Communication Assessment app for formative assessment, summative assessment, and just-in-time coaching of error disclosure communication skills.

## Introduction

Poor communication after a medical injury often leaves patients and families feeling alone, afraid, confused, and more likely to seek redress through malpractice claims [1,2]. One cause of this communication gap cited by both practicing and resident physicians is inadequate training on disclosing harmful medical errors [3,4]. Recently, communication and resolution programs have emerged as a framework to enable clinicians and health care institutions to communicate openly with patients and families, apologize, and offer compensation if an error contributed to patient harm [5]. Communication and resolution programs require clinicians, institutional leaders, and liability insurers to collaborate to provide transparent communication and emotional support for harmed patients. Communication and resolution programs align with recent American Council on Graduate Medical Education mandates that require all trainees to participate in real or simulated disclosure of harm events [6]. However, organizations adopting communication and resolution programs may struggle to prepare physicians for these difficult conversations, in part because of challenges in assessing and improving the specific communication skills required [7].

Traditional methods of assessing physician communication are not suitable for this particular type of task. For example, patient surveys can evaluate actual performance on routine communication, but individual physicians disclose harmful errors infrequently, and these high-stakes discussions are difficult to observe or record. As an alternative to real-world practice, educators often use standardized patients (individuals trained to act as a real patient) and simulated encounters for formative and summative assessments [8]. However, standardized patient exams are logistically intensive, expensive to implement at scale, and lack statistical reliability [9-12]. In addition, it is unknown whether standardized patients or peer physician raters adequately approximate the viewpoint of patients who have experienced medical injury. In particular, physicians’ viewpoints about ideal disclosure content and performance differ from those of patients, which limits physicians’ abilities to assess and coach other physicians’ performance [13,14]. Although feedback would ideally come from harmed patients, researchers have found it difficult to reach this community because providers are reluctant to release details about harmed patients, and because patients hesitate to revisit painful events [15]. To make progress, educators and communication and resolution program leaders need a cost-effective and standardized assessment tool that provides actionable, on-demand, high-volume, and patient-centered feedback about physician communication skills after harm.

The National Board of Medical Examiners recently developed the Video-Based Communication Assessment app as an efficient approach to producing timely, specific, and individual feedback about verbal communication [16]. The Video-Based Communication Assessment app displays brief videos of case vignettes and asks users to audiorecord what they would say next to the patient [17]. Recorded responses are rated by web-based panels of analog patients. Analog patients are untrained raters given the task of listening to and rating their impressions of a medical interaction while assuming the patient perspective [18]. Analog patients are typically laypeople recruited via MTurk [19]; MTurk provides access to a very large, diverse population for survey research, and there is extensive proof that MTurk is an inexpensive, rapid, and high-quality data source [20,21]. Users then receive feedback reports with their individual ratings, comparative data on the user’s cohort, learning points derived from analysis of crowdsourced raters’ comments, and selected highly rated responses from peers. The only study [22] of the Video-Based Communication Assessment app published to date used a variety of 16 typical primary care communication scenarios and found that crowdsourced laypeople can provide high-quality, actionable feedback regarding physician communication skills. Key steps in evaluating the Video-Based Communication Assessment app for error disclosure skill assessment are understanding reliability, educational outcomes, and adoption challenges.

Our aim was to evaluate the reliability of crowdsourced laypeople as raters by comparing their ratings with those of patient advocates who had experienced harm in the course of in their own or a loved one’s medical care. We hypothesized that crowdsourced layperson raters could provide reliable ratings of this specific communication skill, given sufficient panel size.

## Methods

### Overview

This descriptive study is part of a larger project to develop instruments for assessing resident error disclosure skills. With input from experienced attending physicians, we designed and pilot-tested 4 cases specific to the practice of internal medicine. Each case consisted of 3 or 4 vignettes depicting sequential stages in a conversation (for example, initially sharing information about a mistake, responding to a patient’s emotional reaction). We recruited resident physicians at an academic center to use the Video-Based Communication Assessment app. Physicians’ disclosure skills were rated by crowdsourced laypeople recruited on MTurk (Mechanical Turk; Amazon) and by a panel of patient advocates.

### Participants

**Figure 1:**
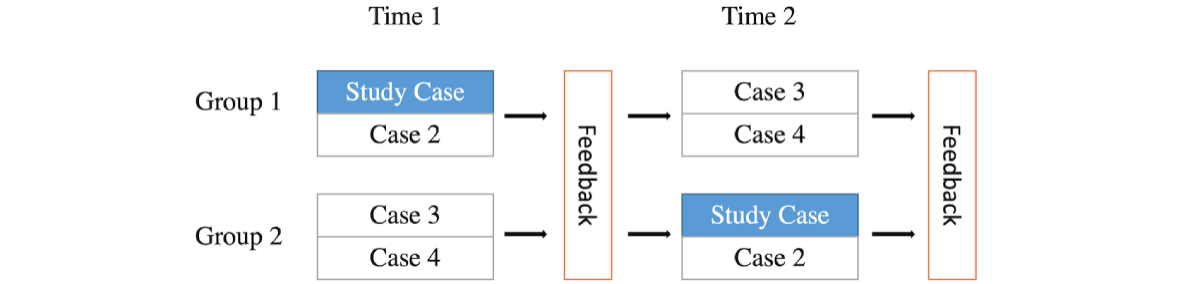
Crossover study design for 21 internal medicine residents using the Video-Based Communication Assessment app at study start (time 1) and approximately 4 weeks later (time 2). The study case in the blue box (breast cancer misdiagnosis) was selected for further study.

We recruited resident physicians in postgraduate years 1 through 3 from the University of Washington academic medical center. We invited all 183 internal medicine residents by email and provided dedicated participation time at a program-wide web-based educational conference (approximate attendance: 40 residents). Residents received a 10-minute orientation to the Video-Based Communication Assessment app and were given class time to participate. Participation was optional. Participants were randomly assigned to 1 of 2 pairs of initial cases to counteract order effects, using a crossover design (Figure 1). After receiving a feedback report, residents were eligible to complete the second 2 cases on their own. Participating residents received a $50 gift card after completing all 4 cases during a 2-month period; however, only 1 case was used in this study.

We used the following inclusion criteria for laypeople: resident of the United States, 18 years or older, and able to speak and read English. Patient advocates were recruited through advertisements with the Patient and Family Advocate Committee of the Collaborative for Accountability and Improvement (a network of health care leaders, attorneys, insurers, and patient advocates who support the development and widespread application of communication and resolution programs). Patient advocates were recruited if they met the following criteria: resident of the United States, 18 years or older, able to speak and read English, not currently or previously employed in health care, and having a personal history of having experienced serious medical injury in their own care or that of a family member. Patient advocates received a US $200 gift card for participation. Crowdsourced raters received variable amounts based on a rate of $0.20 per rating. A crowdsourced rater performing the same total number of ratings as a patient advocate would have received $12.

### Ethics

The University of Washington Institutional Review Board determined that this study was exempt from review for resident, layperson, and patient advocate participants based on its policies, procedures, and guidance [23].

### Video-Based Communication Assessment App

**Figure 2:**
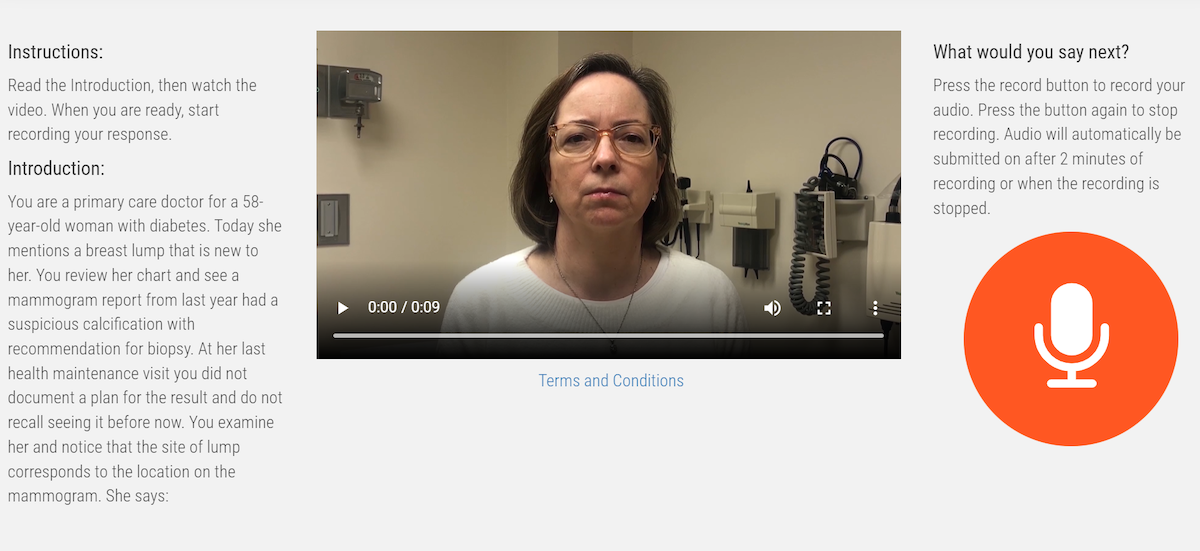
Screenshot from the Video-Based Communication Assessment app displaying a case of delayed diagnosis of breast cancer and the user controls for playing the vignette video and making an audio response to the patient.

The concept and software of the Video-Based Communication Assessment app have been previously described [16]. The app was used to present vignettes, record user responses, and deliver feedback reports (Figure 2). Instead of a single stand-alone vignette, in this study, cases consisted of a linked series of 3 or 4 vignettes to simulate an unfolding conversation. Because a live conversation might not progress in the same manner or sequence, each vignette after the first was accompanied by text declaring what the patient understood at that point.

We used a case that depicted harm resulting from a delayed diagnosis of breast cancer, which is discovered by a primary care doctor just before the patient returns for an office visit (Table 1). This case was chosen because it has 3 segments, rather than 4, which reduced the time and cognitive demands imposed on the small group of patient advocates.

**Table 1:** Text and scenario (spoken by actors in 3 linked vignettes) presented to users (physicians) and raters (laypeople and patient advocates).

Vignette	Situation description (to physician)	Situation Description (to rater)	What the patient says
1	You are a primary care doctor for a 48-year-old woman with diabetes. Today she mentions a breast lump that is new to her. You review her chart and see a mammogram report from last year had a suspicious calcification with recommendation for biopsy. At her last health maintenance visit you did not document a plan for the result and do not recall seeing it before now. You examine her and the site of the lump corresponds to the location on x-ray. She says:	Lorna Smith visits her primary care doctor to evaluate a new breast lump. She figures it isn't anything serious because she had a mammogram last year and never heard about any abnormal results. The doctor examined her and she changed back to regular clothes. She wants to discuss the lump now and says:	“When I didn’t hear from your office about the mammogram, I assumed everything was normal. Was there any sign of this lump on the test last year?”
2	You've told the patient that there were early warning signs of possible breast cancer on her mammogram one year ago. She says:	Lorna has learned that her mammogram last year showed early signs of possible breast cancer, but nothing was done about it. She is feeling panicked and says:	“This is terrible! I’ve never been more frightened…plus you’re telling me that we might have known about it a long time ago!”
3	You've acknowledged how upsetting the error is. The patient now understands that there were early warning signs of possible breast cancer on her mammogram. She says:	Lorna feels like the clinic and her doctor have failed her. She asks:	“How could this happen to me? I feel like I can’t trust anyone anymore. How am I supposed to believe your advice in the future?”

### Data Collection

Resident physicians participated in the video-based communication assessment and provided audio responses to each vignette. All audio responses to a single case were bundled into rating tasks for the raters, comprising 4 physicians’ responses to a case. Raters first completed an audio check and answered questions about demographic characteristics. Raters were asked to read the description of the vignette, view the patient video, listen to each vignette, and rate 6 items (Table 2). Due to the sequential design, we removed raters who did not complete all ratings. We also removed raters who used 2 or fewer response items on the 5-item survey because this may be a sign of inattention and poor rater quality [24]. We defined outliers as raters who reduced the interrater reliability of their task by 0.1 or more.

**Table 2:** Items to assess error disclosure communication skills.

Item	Response options
Overall this provider’s response was	Poor, fair, good, very, good, or excellent
I would feel this provider was accountable for their actions	Not at all, a little, somewhat, very much, or completely
I would feel this provider was being honest about what happened	Not at all, a little, somewhat, very much, or completely
I would feel this provider was sincerely sorry for what happened	Not at all, a little, somewhat, very much, or completely
I would feel the provider understood how I was feeling	Not at all, a little, somewhat, very much, or completely
I would feel this provider cared about me	Not at all, a little, somewhat, very much, or completely
What would you want the provider to say if you were the patient in this situation?	Free text

### Analysis

To create vignette-level scores, ratings were aggregated across all items for each vignette. To compare stringency between groups, we employed a 3×2 repeated measures factorial analysis of variance for vignette (1, 2, 3) and rater (patient advocate, crowdsourced layperson). To create overall assessment scores, we aggregated all vignette-level scores for each user (these continuous scores were derived from ordinal approximations of continuous variables, ie, the mean of Likert-scale responses [25,26]). To determine if an individual physician’s score would be preserved between groups in relation to their peers, we calculated the Pearson correlation.

Generalizability theory utilizes analysis of variance to parse multiple sources of measurement error and estimate reliability under specific conditions [27]. A generalizability analysis was conducted using GENOVA (version 2.1; University of Iowa) to compute variance components for a fully crossed design utilizing a panel of patient advocates [28]. A separate generalizability analysis was conducted using urGENOVA (version 2.1; University of Iowa) to generate variance components for an unbalanced design utilizing crowdsourced layperson raters [29]. In order to determine the optimal design to achieve sufficient reliability, the estimated variance components were used to conduct multiple decision studies to produce G coefficients corresponding to varying numbers of vignettes and raters for each design.

### Patient Advocate Design

To balance consistency and attention span, patient advocates rated batches of 7 physician responses at a time. Batches were block randomized and consisted of physicians’ audio responses to all 3 vignettes. In G-theory, this is referred to as fully crossed design—physician crossed with vignette crossed with rater (p × v × λ).

### Crowdsourced Layperson Design

Crowdsourced laypeople rated a subset of the physicians. Each crowdsourced layperson rated a single batch of 4 physician responses (all 3 vignettes). In G-theory, this is referred to as a rater nested within physician crossed with vignette ((λ:p) × v) design.

## Results

### Participant Demographics

Although 21 internal medicine physicians completed all 3 vignettes, one physician was omitted from analyses because of incomplete ratings); therefore, 20 physicians (male: 6/20, 30%; female: 14/20, 70%), with total of 60 audiorecordings, were rated. The patient advocate panel (n=8; male: 2/8, 25%; female: 6/8,75%) had a median age of 57 years (IQR 53-74.3). Patient advocates reported that it took an average of 116 minutes (SD 62) to rate all 20 cases. A total of 59 crowdsourced laypeople were recruited, but 8 were removed because they did not rate all 3 vignettes in the case, 8 were removed for utilizing 2 or fewer response items, and 1 was deemed to be an outlier; thus, 42 crowdsourced layperson raters were included. Of the 42 crowdsourced raters, 16 (38%) were female; 20 individuals (48%) were between 18 and 34 years old, and 22 (52%) individuals were between 35 and 64 years old.

### Comparing Crowdsourced Laypeople and Patient Advocates:

**Figure 3:**
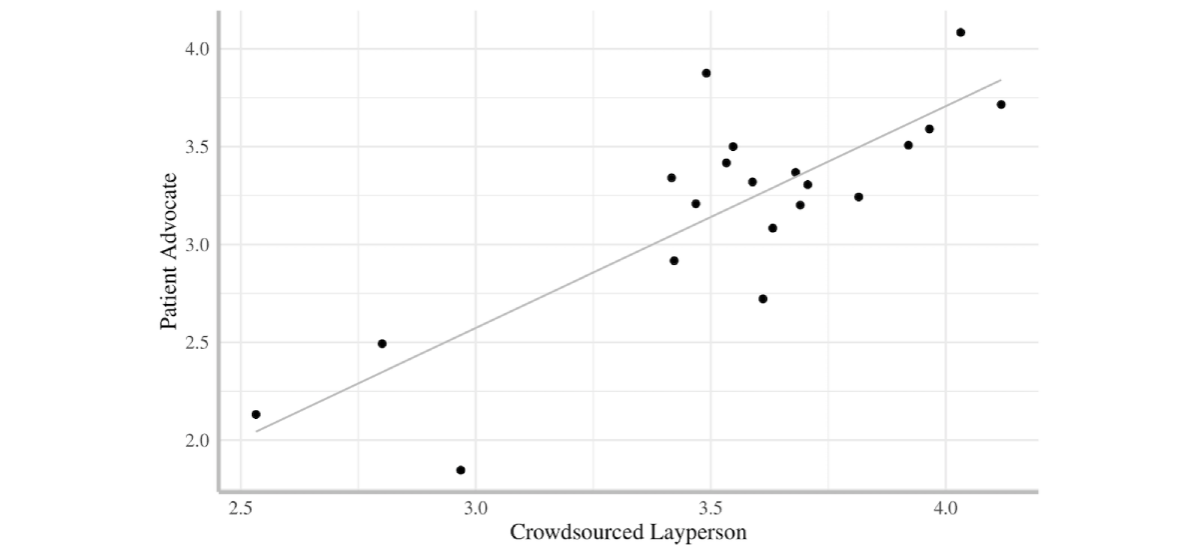
Correlation between ratings of overall communication skill for resident physicians generated by panels of patient advocates and crowdsourced laypeople.

There was a significant overall main effect for rater (*F*_1,19_=24.14, *P*<.001, *d*=0.75)—patient advocates (mean 3.19, SD 0.55) rated communication more stringently than crowdsourced laypeople (mean 3.55, SD 0.40) (Multimedia Appendix 1 and Multimedia Appendix 2). Patient advocate ratings were strongly correlated with crowdsourced layperson ratings (*r*=0.82, *P*<.001) (Figure 3).

### Generalizability

**Figure 4:**
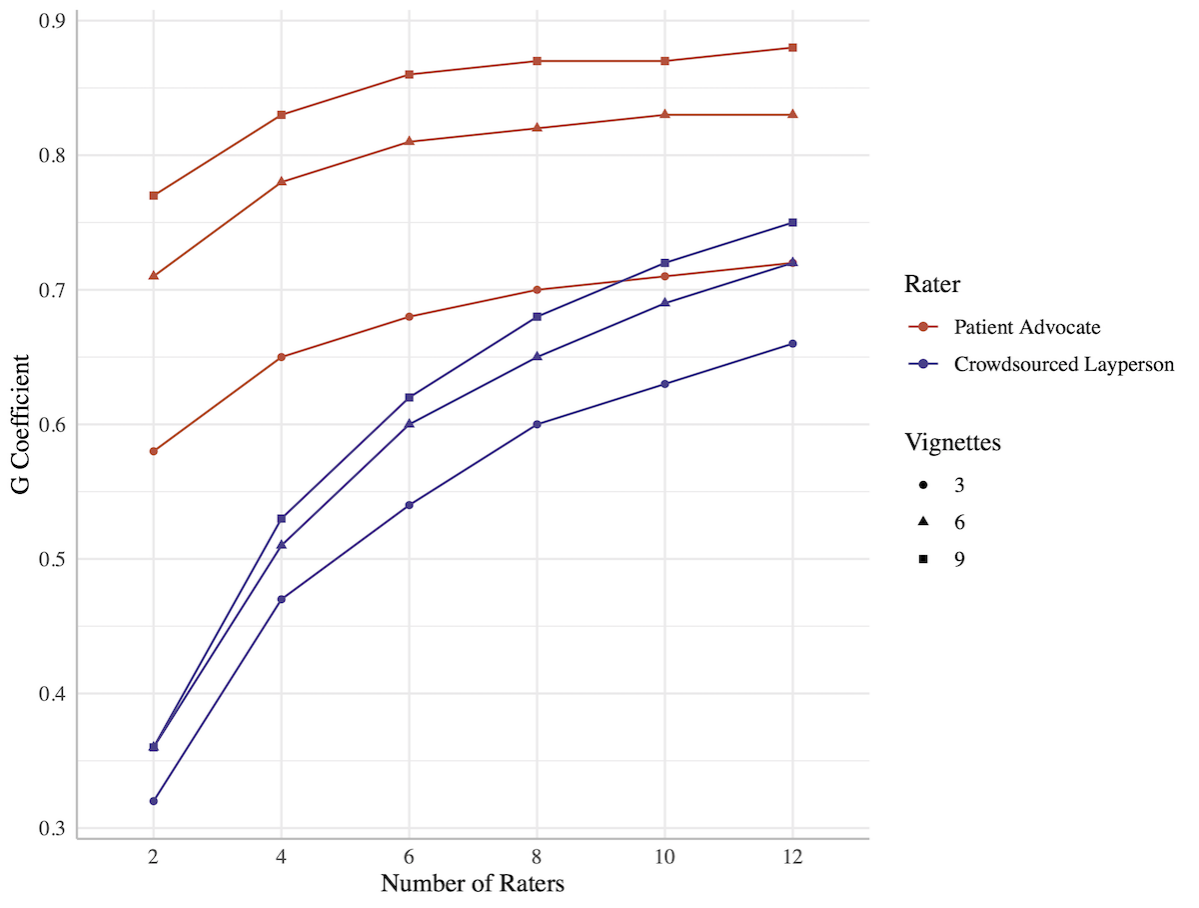
Reliability (G coefficient) models for panels of patient advocates and crowdsourced laypeople, by panel size and number of vignettes rated per user.

Generalizability analysis yielded the variance attributable to each component (Table 3). The G coefficients for 8 raters and 3 vignettes were 0.7 for patient advocates and 0.6 for crowdsourced laypeople. Maintaining 8 raters and increasing the task to 6 vignettes would increase the G coefficients (patient advocates: 0.82; crowdsourced laypeople: 0.65). Increasing the panels to 12 raters for 6 vignettes would increase the G coefficients (patient advocates: 0.83; crowdsourced laypeople: 0.72). Using 12 raters and 9 vignettes would yield G coefficients of 0.88 and 0.75 for patient advocates and crowdsourced laypeople, respectively (Figure 4).

**Table 3:** Generalizability study variance components.

Source of variance			Variance component		Variance percentage
**Patient advocates (p × v ×** **λ** **design)**					
	Physician	0.214		17.979	
	Rater	0.311		26.105	
	Vignette	0.017		1.421	
	Physician × rater	0.008		0.690	
	Physician × vignette	0.210		17.586	
	Rater × vignette	0.012		0.986	
	Residual	0.420		35.232	
**Crowdsourced laypeople ((λ:p) × v design)**					
	Physician	0.121		14.564	
	Vignette	0.007		0.906	
	Rater:physician	0.368		44.402	
	Physician × vignette	0.074		8.952	
	Residual	0.258		31.177	

## Discussion

### Principal Findings

Patient advocates rated communication skills more stringently than crowdsourced laypeople, but the correlation between patient advocates’ ratings and crowdsourced laypeople’s ratings was high. Patient advocates also had higher reliability, but decision studies estimated that panels of crowdsourced laypeople could achieve a G coefficient of 0.75 with 12 raters and 9 vignettes.

These findings demonstrate that crowdsourced laypeople can reliably rate the error disclosure communication skills of physicians using the Video-Based Communication Assessment app. This is encouraging for communication and resolution program leaders and graduate medical educators who require an abundant and affordable pool of raters to support personalized feedback processes in the next generation of physician communication skill training programs. In principle, patient advocates would offer the best possible feedback, but large-scale training efforts would rapidly exhaust the willing and available patient advocate population, given the amount of time that these raters reported spending on this study. Instead, crowdsourced laypeople represent a large and sustainable pool of on-demand raters. Nonetheless, our finding that approximately one-third of crowdsourced laypeople (17/59, 29%) must be removed from analysis to optimize assessment reliability indicates that continuous rater performance monitoring, requirements for raters to complete all vignettes in a series, and a sufficient number of raters would be required for widespread deployment of the Video-Based Communication Assessment app in error disclosure training.

Educators who use the Video-Based Communication Assessment app should understand how crowdsourced raters differ from patient advocates, who represent the gold standard for informed assessment of physician error disclosure skills. Compared with crowdsourced individuals, patient advocates can achieve high reliability with smaller panel sizes and fewer vignettes per physician. This suggests that patient advocates have a common concept of the components of verbal communication that affect the quality of error disclosure and are highly attuned to differences among physicians. Of note, patient advocates assigned lower ratings to resident error disclosure communication than crowdsourced laypeople did. Educators and coaches should recognize that overall scores from crowdsourced laypeople are potentially more generous than those of patients who have experienced harm from medical errors and should note this in reviewing feedback with residents.

### Comparison With Prior Work

The Video-Based Communication Assessment app had been previously only used with groups of stand-alone vignettes [22], but this is the first example of a case with sequential vignettes that simulate a longer conversation. The satisfactory reliability should encourage educators to develop cases for other extended exchanges, such as discussions about goals of care, shared decision-making, or new diagnoses of serious illness. However, our need to sacrifice a subset of ratings by crowdsourced laypeople who had not completed all of the vignettes within a case suggests that longer cases would benefit from a modified approach, such as the use of attention checks or restrictions (eg, a high past task acceptance ratio) [30,31]

Although physician educators have been used to evaluate trainee disclosure skills in a prior study [7], our findings suggest that using faculty as raters would be too costly for large training programs. Based on the time estimates in this study, a residency program with 60 residents, each completing 4 cases, would require an educator to allocate approximately 23 hours to listening and rating audio. Rather than finding 6 to 8 faculty to do this task for a single training session, crowdsourcing laypeople appears to be a more viable and rapid solution.

### Future Directions

This study sets the stage for investigation of use of the Video-Based Communication Assessment app for error disclosure training, for example, for formative assessment (either for self-directed improvement or in conjunction with coaching from a teacher) or summative assessment and in the identification of struggling learners. Although we did not define a threshold for competency, low performers might warrant additional support from residency leaders, including attention on communication performance in other scenarios. Additional areas to explore include whether the tool can be used in undergraduate medical education, continuing medical education, or in just-in-time scenarios (for physicians to practice and receive feedback just before real-life error disclosure). Future studies should investigate the role of different error types (eg, diagnostic or therapeutic), harm severity, physician and patient identity (eg, gender, race), tone, and accent on ratings. The Video-Based Communication Assessment app could be used to understand the efficacy of training interventions and to study the natural history of communication skill development over time. Finally, future studies should also investigate whether error disclosure performance using the Video-Based Communication Assessment app is associated with other safety behaviors encouraged by communication and resolution programs, such as event reporting, root cause analysis, or physician participation in system redesign to prevent future errors.

### Strengths and Limitations

Our work has limitations. We did not assess whether crowdsourced laypeople had personal experience with medical harm and did not measure the amount of time crowdsourced laypeople spent on this evaluation task. Additionally, we recruited patient advocates through their involvement in a national advocacy organization, and their rating behaviors may not generalize to the broader community of patients who have been harmed by care. The convenience sample of patient advocates was not age- and gender-matched to the sample of crowdsourced individuals, and age was not collected as a continuous variable for crowdsourced individuals. The Video-Based Communication Assessment app does not measure nonverbal communication skills, which play an essential role in communication about medical error [32,33]. Finally, this study was conducted using a single case with a breast cancer misdiagnosis and tested with medical residents and may, therefore, not be generalizable to other uses—other unique patient scenarios may require separate validation of crowdsourced laypeople as analog patients. Future research should aim to replicate findings with a more robust sample size.

### Conclusion

Crowdsourced laypeople reliably rated error disclosure skills using the Video-Based Communication Assessment app, although reliably distinguishing high and low performers would require larger panels (9-12 raters) and more vignettes per examinee (9 or more). Fortunately, this is readily achievable in error disclosure curricula. Future studies should focus on the educational outcomes achieved by presenting analog patient feedback to resident physicians about their error disclosure communication skills, and the role of the Video-Based Communication Assessment app in other learner groups or just-in-time scenarios.

## Appendix

Multimedia Appendix 1 Deidentified data of ratings by patient advocates for residents (user) and vignettes (v1,v2,v3).

Multimedia Appendix 2 Deidentified data of ratings by crowdsourced laypeople for residents (user) and vignettes (v1,v2,v3).
